# Transcriptome Analysis Reveals the Role of Sucrose in the Production of *Latilactobacillus sakei* L3 Exopolysaccharide

**DOI:** 10.3390/ijms25137185

**Published:** 2024-06-29

**Authors:** Binbin Wang, Baomei Wu, Min Xu, Kaiyue Zuo, Ye Han, Zhijiang Zhou

**Affiliations:** 1 School of Life Sciences, Shanxi Normal University, Taiyuan 030000, China; wubaomei@sxnu.edu.cn (B.W.); zky3092@163.com (K.Z.); 2 School of Chemical Engineering and Technology, Tianjin University, Tianjin 300072, China; minxu@tju.edu.cn (M.X.); hanye@tju.edu.cn (Y.H.)

**Keywords:** *Latilactobacillus sakei*, synthetic pathway, molecular docking, non-coding RNA, differentially expressed genes

## Abstract

*Latilactobacillus* (*L.*) *sakei* is a species of lactic acid bacteria (LAB) mostly studied according to its application in food fermentation. Previously, *L. sakei* L3 was isolated by our laboratory and possessed the capability of high exopolysaccharide (EPS) yield during sucrose-added fermentation. However, the understanding of sucrose promoting EPS production is still limited. Here, we analyzed the growth characteristics of *L. sakei* L3 and alterations of its transcriptional profiles during sucrose-added fermentation. The results showed that *L. sakei* L3 could survive between pH 4.0 and pH 9.0, tolerant to NaCl (<10%, *w*/*v*) and urea (<6%, *w*/*v*). Meanwhile, transcriptomic analysis showed that a total of 426 differentially expressed genes and eight non-coding RNAs were identified. Genes associated with sucrose metabolism were significantly induced, so *L. sakei* L3 increased the utilization of sucrose to produce EPS, while genes related to uridine monophosphate (UMP), fatty acids and folate synthetic pathways were significantly inhibited, indicating that *L. sakei* L3 decreased self-growth, substance and energy metabolism to satisfy EPS production. Overall, transcriptome analysis provided valuable insights into the mechanisms by which *L. sakei* L3 utilizes sucrose for EPS biosynthesis. The study provided a theoretical foundation for the further application of functional EPS in the food industry.

## 1. Introduction

Lactic acid bacteria (LAB) are mostly labeled “Generally Recognized As Safe” (GRAS) by the US Food and Drug Administration agency and have been widely applied in the long history of fermentation and preservation in the food industry [1]. They produce organic acids, mainly lactic acid, to acidify the raw material. Also, other molecules including ethanol, aroma compounds, bacteriocins, exopolysaccharides (EPSs) and enzymes are yielded to improve the characteristics of the end product [2]. As organic macromolecules and one of the most important secondary metabolites, EPSs have attracted immense scientific interest according to their unique physicochemical characteristics including viscosity, gelation and surface-active properties [3]. Furthermore, LAB EPSs also have beneficial effects on immunoregulatory actions in humans and animals [4]. Most LAB species, including genera of *Fructilactobacillus*, *Lacticaseibacillus*, *Lactobacillus*, *Lactococcus*, *Latilactobacillus*, *Lentilactobacillus*, *Leuconostoc*, *Limosilactobacillus*, *Pediococcus*, *Streptococcus*, and *Weissella*, are so far capable of producing EPSs [5]. However, the structures of these EPSs might be complex and diverse, resulting in different functions.

*Latilactobacillus* (*L.*) *sakei* (formerly known as *Lactobacillus sakei*), assigned to the *Latilactobacillus* genus, is the most commonly studied species according to its sausage fermentation roles and prevalence in the cold storage of raw meat products [6]. EPS yielded by *L. sakei Probio* 65 was reported to possess significantly superoxide dismutase-like activity, while the purified EPS could inhibit alpha-glucosidase and tyrosinase activities with the potential for hyperglycemia control and melanin reduction [7]. These investigations suggested that *L. sakei* EPSs have the potential to be applied in cosmetics, dietary supplements, pharmaceuticals and nutraceuticals.

The yield of EPSs is influenced by several factors, such as carbon sources, especially sugars, in the large-scale production of EPSs [8]. The concentration of EPS produced by *Stenotrophomonas* (*S.*) *maltophilia* and *Brevibacillus* (*B.*) *parabrevis* increased by 65% and 107%, respectively, with 1% of sucrose as the sole carbon source [9]. Meanwhile, maximum EPS yield was also achieved by the fermentation of *L. plantarum* LPC-1 with the addition of sucrose [10].

Transcriptomic analysis is a powerful tool by means of which to investigate the expression of coding RNAs and non-coding RNAs (ncRNA) from one or a group of cells, organs or tissues at a specific developmental stage or in a particular environment [8]. Furthermore, it can reveal the function and potential molecular mechanisms of differentially expressed transcripts which could be anchored to the corresponding sequences in the presence of a reference genome [11]. Studies from *S. thermophilus* IMAU20561 cultivated in different nitrogen sources suggested that the yield of EPS was associated with the pathways of amino acid biosynthesis, glycolysis, the phosphotransferase system, and fructose and mannose metabolism [12]. Furthermore, another investigation implied that carbohydrate metabolism was conducive to high EPS production [13]. Thus, transcriptome sequencing is an effective method by means of which to study the influence of internal and external factors on the EPS produced by LAB.

In our previous research, *L. sakei* L3 was isolated from the Hubei sausage and able to produce EPS with high yield during sucrose-added fermentation [14,15]. However, the biosynthetic relationships between *L. sakei* L3 EPS production and sucrose as a carbon source are still unclear. Therefore, the method of transcriptome sequencing was used to explore the aforementioned relationships based on the obtained data of differentially expressed genes (DEGs) and ncRNAs in this study. Additionally, the basic growth characteristics of *L. sakei* L3 were also investigated. This research provides a theoretical basis for the further mechanistic application of *L. sakei* L3 EPS production.

## 2. Results and Discussion

### 2.1. Growth Characteristics of L. sakei L3

**Figure 1:**
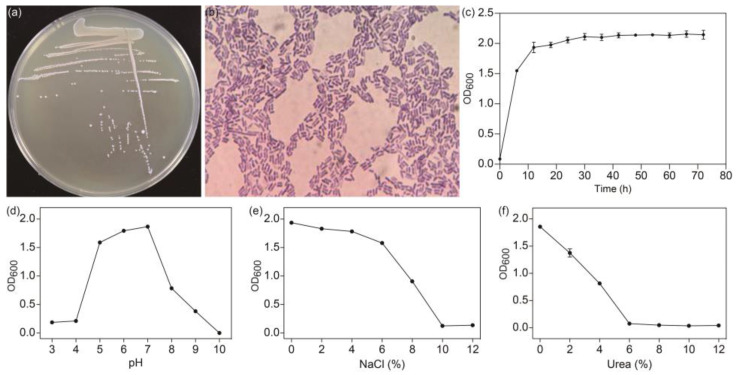
The morphology and growth characteristics of *L. sakei* L3. (**a**) The morphology of colonies; (**b**) Gram’s staining; (**c**) The growth curve of *L. sakei L3*; (**d**) pH tolerance analysis; (**e**) NaCl tolerance analysis; (**f**) Urea tolerance analysis. OD_600_: Optical density measurements at a wavelength of 600 nm.

As shown in Figure 1a, the *L. sakei* L3 colonies showed circular morphologies with a moist surface and smooth edges. Meanwhile, *L. sakei* L3 was a gram-positive organism according to Gram’s staining method (Figure 1b). Subsequently, the growth curve of *L. sakei* L3 was measured in the MRS liquid medium with a 2% (*v*/*v*) inoculum concentration and static culture. According to Figure 1c, *L. sakei* L3 arrived at the stationary phase around 15 h after inoculation. Based on the obtained time point, the tolerance of *L. sakei* L3 to pH, NaCl and urea was explored. The results shown in Figure 1d–f demonstrate that *L. sakei* L3 was suitable for survival between pH 4.0 and pH 9.0, tolerant to NaCl (<10%, *w*/*v*) and urea (<6%, *w*/*v*), while probiotic strain *Lactococcus lactis* Gh1 was tolerant to pH 3.0, which was more suitable than our aforementioned pH, and NaCl (≤4.0%, *w*/*v*), which was lower than the maximum tolerance NaCl concentration of *L. sakei* L3 [16]. Acid tolerance was one of the most important characteristics of probiotics, whereby they could live in the acid environment to promote human health [17]. NaCl tolerance could enable the bacteria to withstand the high osmotic pressures to maintain the stability of strains’ inner physicochemical properties [16,18], improving the growth of probiotics in the human body. Additionally, it was reported that a candidate probiotic microbe of *Enterococcus faecium* R8 was able to hydrolyze urea [19], which was similar with *L. sakei* L3, indicating it might play certain roles in urea-related diseases. Overall, *L. sakei* L3 might possess potential application prospects.

**Figure 2:**
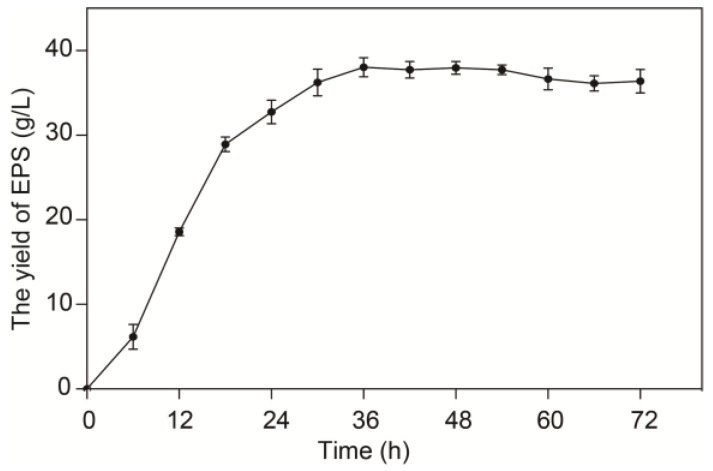
Time-course accumulation of EPS during fermentation of *L. sakei* L3.

In addition, *L. sakei* L3 was able to utilize sucrose to produce EPS according to our previous report [14]. Consequently, time-course accumulation of EPS was further analyzed during fermentation in the MRS-S medium. Figure 2 illustrates that the yield of EPS reached maximum level after 36 h of fermentation and subsequently exhibited stabilization. In order to explore the transcriptional responses of *L. sakei* L3 to sucrose during fermentation, the transcriptome analysis was conducted based on the time point of 36 h.

### 2.2. Sequencing Quality Assessment and Comparison with Reference Genomes

The transcriptome of *L. sakei* L3 was profiled after 36 h of inoculation using the Illumina HiSeq platform. 24.88 ± 0 Mb raw data were separately obtained from MRS and MRS-S groups (Table 1). After removing the adaptor and low-quality sequences, the clean reads from the two groups were 24.28 ± 0.07 Mb and 24.21 ± 0.16 Mb, respectively. Meanwhile, clean Q20 and Q30, respectively, were >98% and >97%, and the clean read ratio was >95% (Table 1), indicating the sequencing quality of the samples were accurate and reliable with a low base error rate. These results implied that the obtained sequencing data could be used for further biological analysis.

The filtered clean reads were compared with the reference genome of *L. sakei* 23K [20], which is an important model organism of *L. sakei* species [20]. As shown in Table 2, the comparison rate was >78%, and the data could satisfy the experimental requirements of subsequent analysis.

### 2.3. Screening of DEGs between MRS and MRS-S Groups

**Figure 3:**
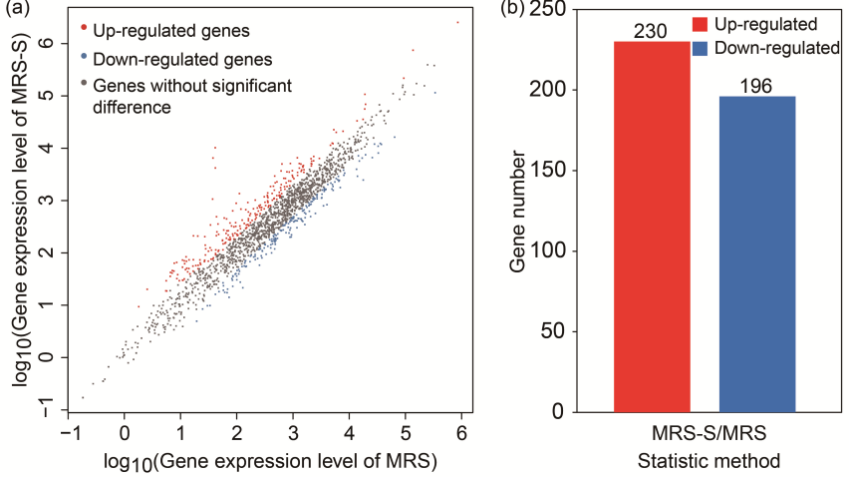
Gene expression level analysis of *L. sakei* L3 between MRS and MRS-S groups. (**a**) Scatter plot of DEGs; (**b**) Number of up-regulated and down-regulated DEGs.

A total of 426 DEGs (|log_2_(fold-change)| ≥ 1, *p* < 0.05) were identified in the MRS-S groups relative to MRS groups (Figure 3a). Among these DEGs, 230 and 196 genes were up-regulated and down-regulated, respectively (Figure 3b). These results indicated that the gene expression profiles of *L. sakei* L3 was significantly varied with the addition of sucrose during the fermentation.

### 2.4. GO Annotations Analysis

**Figure 4:**
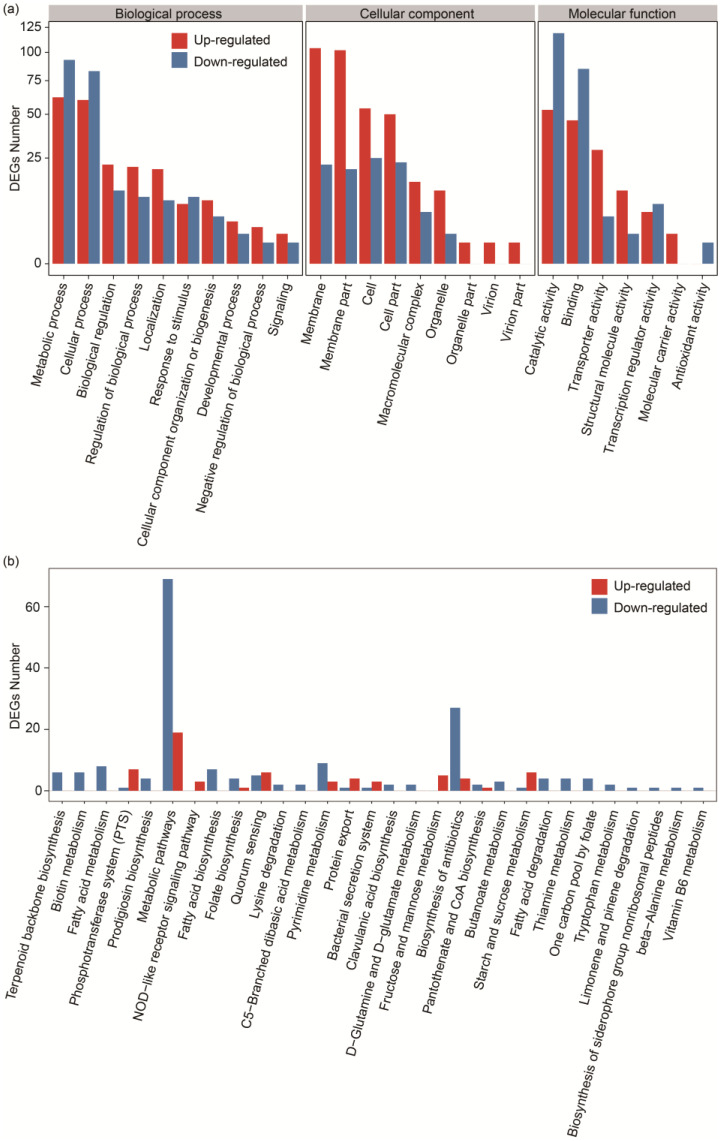
GO annotation and KEGG pathway analysis of DEGs of *L. sakei* L3 during fermentation. (**a**) GO annotations of DEGs; (**b**) Pathway functional enrichment according to up- and down-regulated genes.

To further reveal the response of *L. sakei* L3 to sucrose, DEGs were subjected to GO functional annotation. Three categories, biological process (BP), cellular component (CC) and molecular function (MF), were divided by the GO database (Figure 4a). The obtained DEGs were mainly concentrated in the metabolic process (155), cellular process (143), biological regulation (34) and regulation of biological process (31) of the BP category, in the membrane (126), membrane part (122), cell (79) and cell part (73) of the CC category, and in the catalytic activity (172), binding (131) and transporter activity (34) of the MF category. Moreover, among the aforementioned GO terms, the number of up-regulated DEGs in metabolic process, cellular process, catalytic activity and binding terms were less than the number of down-regulated genes, while DEGs in other terms generally showed the opposite trend.

### 2.5. KEGG Pathway Analysis

The KEGG database provides important information on biological systems related to molecular interaction and reaction networks [21], which played vital roles in understanding complex genetic and biological behaviors during omics analysis of differently treated samples. After GO annotation analysis, the KEGG pathway analysis of the obtained DEGs was carried out between the MRS and MRS-S groups. According to Figure S1, the DEGs were assigned to different KEGG metabolic pathways which were divided into five categories: cellular processes, environmental information processing, genetic information processing, metabolism and organismal systems. The DEGs were mainly clustered in the carbohydrate metabolism (33), metabolism of cofactors and vitamins (26), nucleotide metabolism (22) and membrane transport (21).

Thirty of the most enriched pathways between the MRS and MRS-S groups are shown in Figure 4b. The number of up-regulated DEGs in the phosphotransferase system (PTS), NOD-like receptor signaling pathway, quorum sensing, protein export, bacterial secretion system, fructose and mannose metabolism and starch and sucrose metabolism was greater than the number of down-regulated genes, while DEGs in other enriched pathways exhibited the opposite trend.

### 2.6. Critical DEG Analysis under Sucrose-Added Fermentation

#### 2.6.1. The Positive Regulation of Genes Involved in Sucrose Metabolism in the MRS-S Group

**Figure 5:**
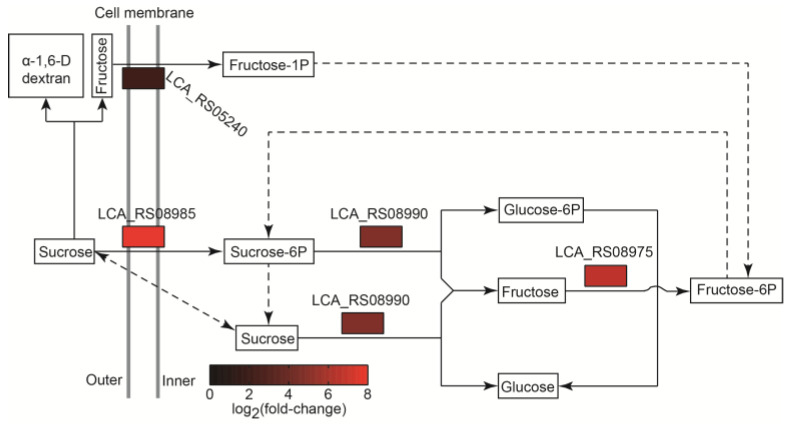
The pathway analysis of sucrose metabolism in *L. sakei* L3 during fermentation.

Sucrose plays important roles in the production of intracellular polysaccharide and EPS during fermentation [22] and can also degrade to glucose and fructose in suitable conditions [23]. Our previous study suggested that *L. sakei* L3 could produce EPS, an α-1,6 dextran, in the sucrose-added medium [14]. According to Figure 5 and Table 3, the PTS-S (LCA_RS08985) and PTS fructose transporter subunit IIC (PTS-F, LCA_RS05240), assigned to the phosphotransferase system (PTS) relating to the transport and metabolism of sugars from the extracellular to the intracellular sides of cells [24], were significantly up-regulated, indicating sucrose could be degraded into fructose during EPS production and simultaneously transported into cells during fermentation. Accordingly, sucrose-6-phosphate hydrolase (LCA_RS08990), which converts sucrose-6-phosphate to fructose and glucose-6-phosphate [25], and fructokinase (LCA_RS08975), which phosphorylates fructose to fructose-6-phosphate [26], were significantly up-regulated (Figure 5 and Table 3). The products yielded by the two enzymes could meet the energy and material metabolisms of *L. sakei* L3 as well as being further catalyzed to produce sucrose to synthesize EPS (Figure 5).

**Figure 6:**
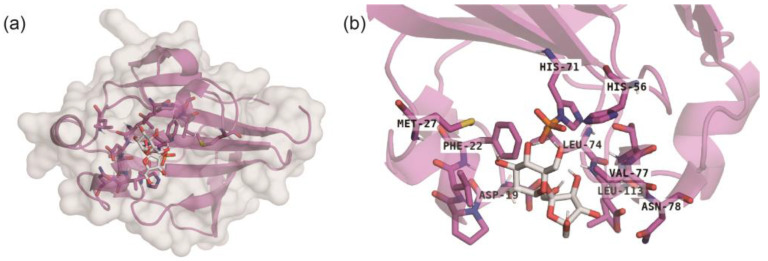
Molecular docking of sucrose and PTS-S. (**a**) 3D-structural modeling of sucrose and PTS-S; (**b**) The interactions of critical amino acids with sucrose.

In order to explore binding models between sucrose and PTS-S in the molecular level, molecular docking was conducted. Sucrose was bound to the active pocket of the PTS-S in a compact conformation (Figure 6a), and the active pocket was formed by the amino acids of Asp^19^, Phe^22^, Met^27^, His^56^, His^71^, Leu^74^, Val^77^, Asn^78^ and Leu^113^ with van der Waals interactions (Figure 6b). Among these amino acids, hydrophobic interactions were found between sucrose and Phe^22^, Met^27^, Leu^74^, Val^77^ and Leu^113^ (Figure 6b), which resulted in the formation of stable sucrose and PTS-S complexes to favor the intracellular transport of sucrose during fermentation.

#### 2.6.2. The Negative Regulation of Genes Involved in the Uridine Monophosphate (UMP) Pathway in the MRS-S Group

**Figure 7:**
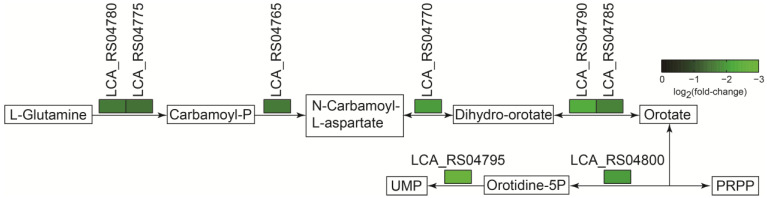
The analysis of the UMP synthetic pathway in *L. sakei* L3 during fermentation.

Pyrimidine nucleotide biosynthesis consists of nucleoside salvage and de novo pathways resulting in the generation of UMP [27,28], which is composed of uracil base, ribose sugar and a phosphoester group [29]. UMP is the precursor of all other pyrimidine nucleotides; the de novo synthesis of UMP was previously reported to be a common biosynthetic pathway in all organisms and essential for the synthesis of nucleic acids [30]. The de novo pathway includes six steps that utilize glutamine, aspartate, bicarbonate and glucose to produce UMP [28]. During sucrose-added fermentation, eight enzymes modulating the synthesis of UMP in the six steps were significantly down-regulated (Figure 7 and Table 3). Among the eight enzymes, carbamoyl-phosphate synthase consisting of small (LCA_RS04775) and large (LCA_RS04780) subunits could catalyze the synthesis of the carbamoyl-phosphate from glutamine or ammonia, bio-carbonate and ATP [31], which initiates the synthesis of UMP. Meanwhile, orotidine-5′-phosphate decarboxylase (LCA_RS04795) catalyzes the conversion of orotidine-5′-phosphate to UMP, which is the last step in the de novo biosynthesis of pyrimidine nucleotides [32]. Consequently, the inhibition of UMP synthesis in the *L. sakei* L3 during sucrose-added fermentation would finally repress the biosynthesis of the DNA or RNA, thereby decreasing the bacteria growth. These results were similar to a previous report of transcriptome analysis on the effect of soybean protein and peptides on *Lacticaseibacillus rhamnosus* Lra05 [33]. Consequently, the growth inhibition of *L. sakei* L3 could be beneficial for EPS synthesis utilizing sucrose.

#### 2.6.3. The Negative Regulation of Genes Involved in the Fatty Acid Synthetic Pathway in the MRS-S Group

**Figure 8:**
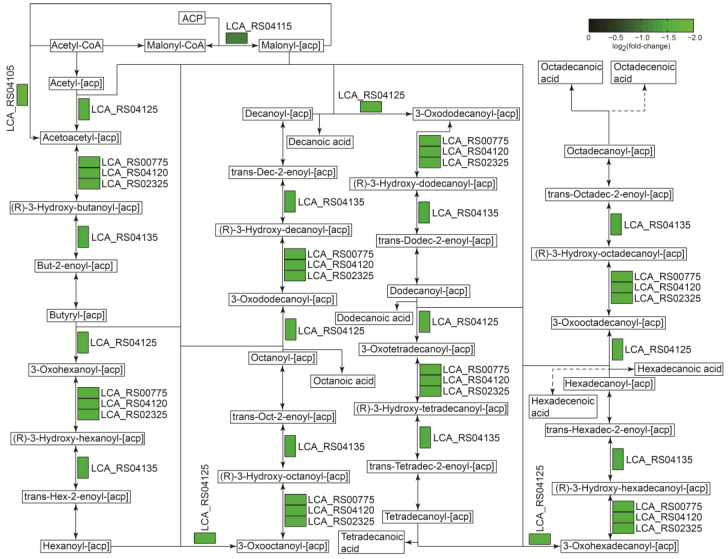
The analysis of the fatty acid synthetic pathway in *L. sakei* L3 during fermentation.

Fatty acids play important roles in membrane lipid homeostasis and the energy metabolism in all organisms [34], and their degradation and biosynthesis pathway must be rigorously switched on and off according to the external environment and internal physicochemical changes of organisms. The fatty acid biosynthesis pathway among bacteria is generally highly conserved and requires acetyl-CoA as raw material and various enzymes [35]. During the fermentation of *L. sakei* L3 in the MRS-S medium, seven genes in the fatty acid biosynthesis pathway were significantly down-regulated (Figure 8 and Table 3). Among the seven genes, the gene encoding a hypothetical protein (LCA_RS04115) was assigned to catalyze malonyl-CoA and ACP to generate malonyl-ACP, which was subsequently condensed with acetyl-CoA to generate acetoacetyl-ACP, known as the first step in fatty acid synthesis [36,37], while another five genes functioned in different steps to regulate fatty acid synthesis (Figure 8). These results indicated that fatty acid biosynthesis was significantly reduced in *L. sakei* L3 during sucrose-added fermentation, which might be beneficial for EPS production.

### 2.7. The Analysis of ncRNA and Its Target Genes during Sucrose-Added Fermentation

ncRNAs, which are generated from specific transcriptional units or existing RNA, are important regulatory molecules including small ncRNAs and long ncRNAs and can modulate a myriad of biological processes through binding target genes, proteins, and so forth [38,39]. In order to explore whether ncRNAs were involved in the sucrose-added fermentation of *L. sakei* L3 to participate in EPS production, the differentially expressed profiles of ncRNAs were analyzed.

**Figure 9:**
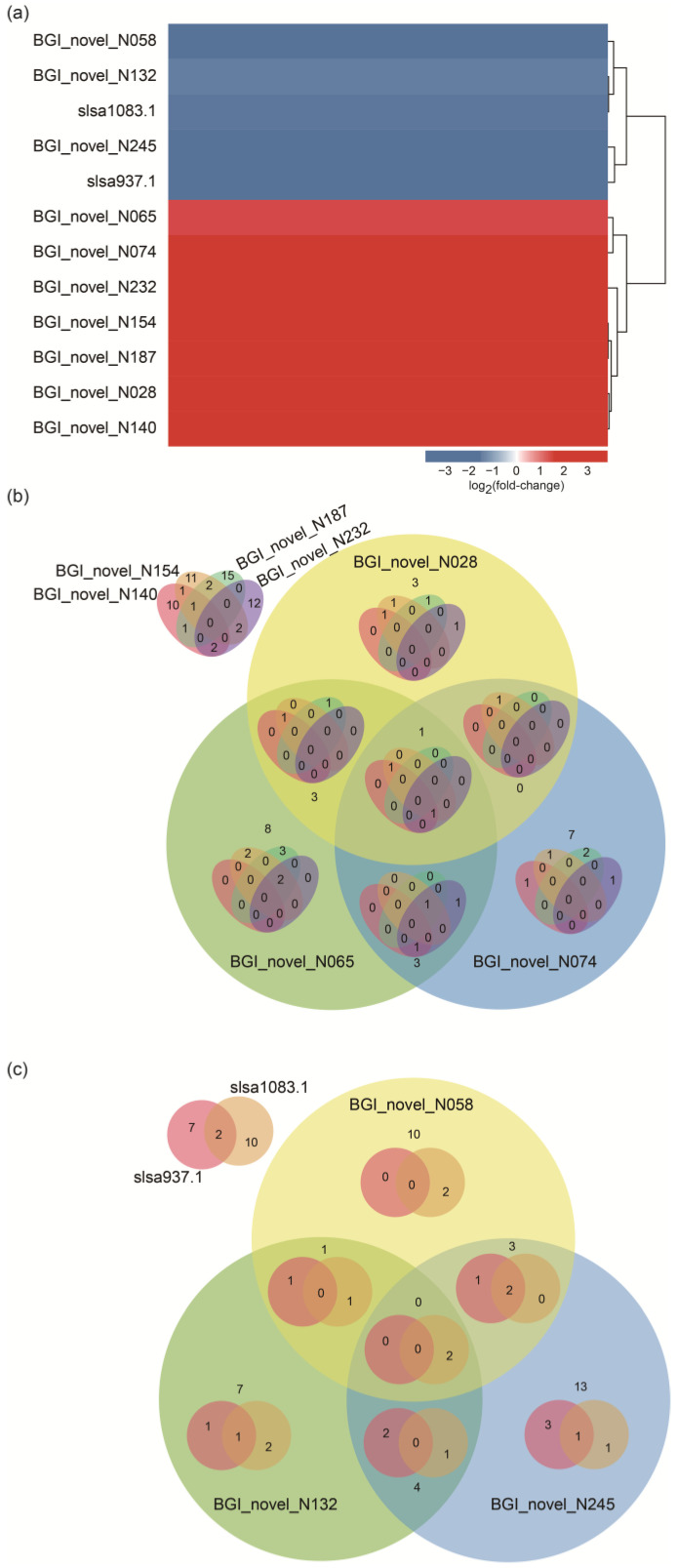
The analysis of differentially expressed ncRNAs and their coregulated target genes in *L. sakei* L3 during fermentation. (**a**) Differentially expressed ncRNAs; (**b**) Coregulated target genes modulated by seven up-regulated ncRNAs; (**c**) Coregulated target genes modulated by five down-regulated ncRNAs.

A total of 12 differentially expressed ncRNAs (|log_2_(fold-change)| ≥ 1, *p* < 0.05) were screened in the MRS-S groups relative to the MRS groups (Figure 9a). Seven and five ncRNAs were significantly up-regulated and down-regulated, respectively. Subsequently, the target genes of 12 ncRNAs were analyzed based on the abovementioned 426 DEGs. The results showed that 49 up-regulated and 92 down-regulated DEGs regulated by these ncRNAs were found in the MRS-S group (Figure S2 and Table S1). Further analysis suggested that one target gene might be modulated by diverse members of the 12 ncRNAs (Figure S2 and Table S1), such as PTS-F, which was coregulated by BGI_novel_N074 and BGI_novel_N187. Therefore, the target genes coregulated by the seven up-regulated and down-regulated ncRNAs, respectively, were investigated using the Venn method. As shown in Figure 9b and Table 3 and Table S1, LCA_RS05480 encoding a folylpolyglutamate synthase (FPGS) was coregulated by six up-regulated ncRNAs, and LCA_RS09375 encoding a TVP38/TMEM64 family protein (TTP) was coregulated not only by five up-regulated ncRNAs but also by four down-regulated ncRNAs (Figure 9c and Table 3 and Table S1). LCA_RS03600 encoding a glycerate kinase was also coregulated by four down-regulated ncRNAs (Figure 9c and Table 3 and Table S1). Intriguingly, all of the three target genes were down-regulated in the MRS-S group (Table 3).

**Figure 10:**
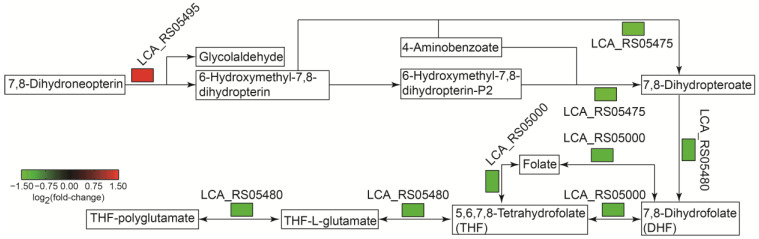
The analysis of the folate synthetic pathway in *L. sakei* L3 during fermentation.

FPGS is a key enzyme involved in folate metabolism to catalyze the formation of polyglutamates from folates [40,41]. The down-regulation of FPGS suggested that folate biosynthesis might be affected by sucrose-added fermentation. Folates play important roles in critical metabolisms, including metabolisms of nucleotides, pantothenic acid and amino acids, as one carbon pool. As shown in Figure 10, folate biosynthesis was significantly down-regulated to reduce the formation of polyglutamates as well as nucleotides such as the aforementioned UMP, indicating that *L. sakei* L3 inhibited several critical metabolisms to enhance the yield of EPS.

**Figure 11:**
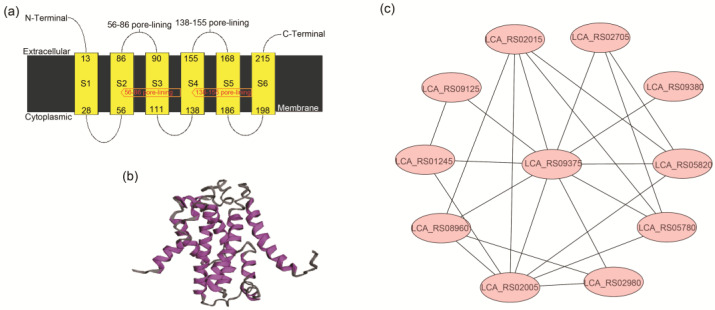
Bioinformatic analysis of TTP encoded by LCA_RS09375 in *L. sakei* L3. (**a**) Membrane-spanning domain prediction of TTP; (**b**) Three-dimensional structure analysis of TTP; (**c**) Interaction network analysis of TTP. LCA_RS09125: Cell surface protein, LCA_RS01245: DUF2798 domain-containing protein, LCA_RS08960: Cell surface protein, LCA_RS02005: DUF4811 domain-containing protein, LCA_RS02980: Glycerophosphoryl diester phosphodiesterase, LCA_RS05780: Peptidase M23, LCA_RS05820: Peptidase M23, LCA_RS09380: Hypothetical protein, LCA_RS02705: Membrane protein, LCA_RS02015: MerR family transcriptional regulator.

Additionally, TTP was assigned to membrane protein [42]. Our result of bioinformatic prediction about TTP encoded by LCA_RS09375 in *L. sakei* L3 was consistent with the report. Figure 11a,b illustrated that TTP contained six membrane-spanning domains with N- and C-terminals outside the membrane. TTP could interact with cell surface proteins (LCA_RS09125 and LCA_RS08960), membrane protein (LCA_RS02705), domain-containing proteins (DUF2798, LCA_RS01245 and DUF4811, LCA_RS02005), glycerophosphoryl diester phosphodiesterase (LCA_RS02980), peptidases (LCA_RS05780 and LCA_RS05820) and a MerR family transcriptional regulator (LCA_RS02015) (Figure 11c). These results suggested that TTP played important roles in molecular transport. Consequently, the down-regulation of TTP affected the transmembrane transport of specific materials in *L. sakei* L3 during sucrose-added fermentation.

Furthermore, glycerate kinase catalyzes the conversion of glycerate to 2-phosphoglycerate or 3-phosphoglycerate to not only modulate the metabolisms of serine and glycolic acid but also become involved in the Entner–Doudoroff pathway [43,44,45,46]. The down-regulation of glycerate kinase in *L. sakei* L3 during sucrose-added fermentation indicated that *L. sakei* L3 utilized effective substrates to synthesize the EPS to inhibit other metabolisms.

### 2.8. Validation of Typical DEGs Using RT-qPCR

**Figure 12:**
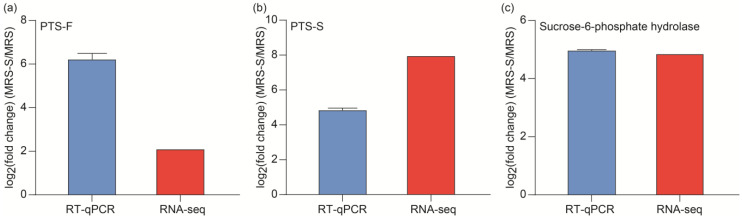
Validation of selected DEGs from sucrose metabolism using RT-qPCR. (**a**) PTS-F (LCA_RS05240); (**b**) PTS-S (LCA_RS08985); (**c**) sucrose-6-phosphate hydrolase (LCA_RS08990).

To verify the reliability of the obtained transcriptome data, three genes related to sucrose metabolism were selected to conduct the RT-qPCR analysis. As shown in Figure 12, the three genes showed good consistency of RT-qPCR results and transcriptome data, despite some differences in the expression levels. The result indicted that the transcriptome analysis was reliable and accurate.

## 3. Materials and Methods

### 3.1. Bacterial Strains and Culture Conditions

*L. sakei* L3 was isolated from fermented Hubei sausage (Wuhan, Hubei Province, China) using de Man–Rogosa–Sharpe (MRS) agar medium containing 50 g/L sucrose (MRS-S) by our laboratory [14], and used throughout this study. *L. sakei* L3 was cultivated on the MRS agar medium to obtain an individual bacterial colony based on the previous report [14]. Then, the colony was cultivated in a 250 mL flask containing 50 mL of seed broth medium at 30 °C for 15 h. *L. sakei* L3 was incubated at 2% inoculation rate (*v*/*v*) in MRS and MRS-S liquid media to analyze the growth and EPS yield, respectively. The growth medium was cultivated at 30 °C statically and sampled every six hours to detect the OD_600_ values, while the EPS yield medium was cultivated on a rotary shaker at 110 rpm and also sampled every six hours to extract EPS according to the method of phenol sulfuric acid [47]. Three replicates were set for each sample. All the microbiological media were purchased from Jiangtian Chemical Technology Co., Ltd., Tianjin, China.

### 3.2. Biological Characteristics Analysis of L. sakei L3

#### 3.2.1. Morphological Analysis

In order to assess the Gram stain status and morphological features of *L. sakei* L3, Gram staining was carried out based on previous protocols [48].

#### 3.2.2. Biochemical Characteristics Tests

The growth conditions of *L. sakei* L3 at different pHs (pH 3.0 to pH 10.0), NaCl (AOBOX Biotechnology Co., Ltd., Beijing, China) concentrations (2.0%, 4.0%, 6.0%, 8.0% and 10.0%, *w*/*v*) and urea (AOBOX Biotechnology Co., Ltd., Beijing, China) concentrations (2.0%, 4.0%, 6.0%, 8.0%, 10.0% and 12.0%, *w*/*v*) were performed with a 2% inoculation rate (*v*/*v*) of seed broth medium in the MRS liquid medium. The inoculation was conducted at 30 °C statically for 15 h. The OD_600_ values were subsequently collected. Three replicates were set for each sample.

### 3.3. Transcriptomic Sequencing Analysis

#### 3.3.1. Sample Preparation

100 mL of *L. sakei* L3 suspensions which were cultured for 36 h based on the EPS yield analysis in the MRS or MRS-S medium were centrifuged at 4 °C for 5 min (6000 r/min); the obtained *L. sakei* L3 was washed with sterile water, and then quickly frozen in the liquid nitrogen. Two biological replicates were performed. The transcriptome sequencing was conducted with the Illumina HiSeq platform by BGI-Shenzhen, China.

#### 3.3.2. Transcriptome Sequencing Data Processing

Quality control was conducted on raw sequencing data through removing reads with low quality, adapters and more than 5% of N bases to obtain high-quality clean reads. Then, they were aligned to the reference genome of *L. sakei* 23K using HISAT software v2.0.1-beta [49]. The differentially expressed genes (DEGs) and ncRNAs (length between 100 and 600 bps) were screened with the criteria of adjust *p*-value < 0.05 and |log_2_(fold-change)| ≥ 1.

#### 3.3.3. Functional Analysis of DEGs

Gene Ontology (GO) functional annotation analysis and Kyoto Encyclopedia of Genes and Genomes (KEGG) pathway annotation analysis were performed for DEGs, and the corrected *p*-value < 0.05 was considered to be significantly enriched.

#### 3.3.4. Molecular Docking Analysis

Molecular docking was performed using Autodock vina 1.1.2 software [50]. The structures of sucrose and PTS sucrose transporter subunit IIABC (PTS-S) encoded by LCA_RS08985 were firstly obtained, and PDBQT files were finally acquired after the optimization of force-field energy by AutodockTools 1.5.6 software [51,52].

#### 3.3.5. ncRNA Target Genes and Interaction Network Analysis

The target genes of differentially expressed ncRNAs were predicted with RNAplex software v2.3.4 [53]. The obtained target genes and ncRNAs were subsequently used to construct the interaction network by means of Cytoscape software 3.7.1 [54]. Additionally, the coregulated target genes of up- and down-regulated ncRNAs were also analyzed using Vennpainter software 1.2.0 [55], respectively.

#### 3.3.6. Bioinformatic Analysis of TTP Encoded by LCA_RS09375

The membrane-spanning domain prediction and three-dimensional structure analysis of TTP encoded by LCA_RS09375 were performed using PSIPRED software version 4 [56]. The interaction network of TTP was firstly analyzed by means of STRING software v11 [57], and then reconstructed with Cytoscape software 3.7.1 [54].

#### 3.3.7. Real-Time Fluorescence Quantitative PCR (RT-qPCR) Verification of DEGs

Three genes from the sucrose metabolism were analyzed using RT-qPCR to evaluate the validity of the transcriptome date. After total RNA extraction from *L. sakei* L3 cultivated in the MRS and MRS-S media for 36 h, cDNA was obtained using a PrimeScript™ RT reagent Kit with gDNA Eraser (Takara Biomedical Technology Co., Ltd., Beijing, China). RT-qPCR analysis was performed based on the instructions of SYBR^®^ Premix Ex Taq™ II (Tli RNaseH Plus), ROX plus (Takara Biomedical Technology Co., Ltd., Beijing, China) on the ABI7500 Real Time PCR system (Applied Biosystems, Inc., Waltham, MA, USA) with the primers of selected DEGs (Table S2). The process was repeated three times for each DEG. The fold change of the DEGs was quantified by the 2^−ΔΔCt^ method.

## 4. Conclusions

In this study, we used transcriptomic technology to investigate the role of sucrose for EPS production in *L. sakei* L3. The results showed that sucrose-adding fermentation of *L. sakei* L3 significantly enhanced the sucrose metabolism and inhibited UMP, fatty acids and folate synthetic pathways, indicating that *L. sakei* L3 promoted the EPS yield through down-regulating other metabolic pathways, which was beneficial for the flow of substance and energy towards the EPS synthesis. Moreover, the enhancement of sucrose metabolism not only satisfied the basic growth needs of *L. sakei* L3, but also further facilitated the biosynthesis of the EPS. Additionally, ncRNAs and their target genes were also regulated by sucrose and played important roles in EPS production. PTS-F was coregulated by two up-regulated ncRNAs of BGI_novel_N074 and BGI_novel_N187, indicating ncRNAs were involved in sucrose metabolism. Overall, transcriptome analysis provided valuable insights into the mechanisms by which *L. sakei* L3 utilizes sucrose for EPS biosynthesis. This study provides the foundation for further exploring structure–activity relationships of *L. sakei* L3 EPS to obtain functional foods with different molecular weights according to technologies of synthetic biology.

## Figures and Tables

**Table 1:** Clean read quality metrics.

Sample	Total Raw Reads (Mb)	Total Clean Reads (Mb)	Total Clean Bases (Gb)	Clean Reads Q20 (%)	Clean Reads Q30 (%)	Clean Reads Ratio (%)
MRS	24.88 ± 0	24.28 ± 0.07	2.43 ± 0.01	98.98 ± 0.08	96.92 ± 0.20	97.58 ± 0.30
MRS-S	24.88 ± 0	24.21 ± 0.16	2.42 ± 0.01	99.01 ± 0.01	96.99 ± 0.02	97.29 ± 0.63

**Table 2:** Comparison between reads and reference genome.

Sample	Total Clean Reads (Mb)	Total Mapping (%)	Unique Mapping (%)
MRS	24.28 ± 0.07	78.36 ± 0.81	62.88 ± 6.99
MRS-S	24.21 ± 0.16	82.13 ± 0.08	48.15 ± 2.00

**Table 3:** DEGs involved in critical metabolism pathways and the ncRNA regulatory network.

Gene ID	log_2_(Fold-Change)	Description
LCA_RS05240	2.09	PTS fructose transporter subunit IIC
LCA_RS08975	6.66	Fructokinase
LCA_RS08985	7.95	PTS sucrose transporter subunit IIABC
LCA_RS08990	4.84	Sucrose-6-phosphate hydrolase
LCA_RS04780	−1.55	Carbamoyl-phosphate synthase large chain
LCA_RS04775	−1.53	Carbamoyl-phosphate synthase small subunit
LCA_RS04765	−1.58	Aspartate carbamoyltransferase
LCA_RS04770	−2.03	Dihydroorotase
LCA_RS04790	−2.27	Dihydroorotate dehydrogenase
LCA_RS04785	−1.73	Dihydroorotate dehydrogenase electron transfer subunit
LCA_RS04800	−1.96	Orotate phosphoribosyltransferase
LCA_RS04795	−2.85	Orotidine-5′-phosphate decarboxylase
LCA_RS04115	−1.12	Hypothetical protein
LCA_RS04105	−1.79	3-oxoacyl-ACP synthase III
LCA_RS04125	−1.57	Beta-ketoacyl-[acyl-carrier-protein] synthase II
LCA_RS00775	−1.6	Oxidoreductase
LCA_RS04120	−1.65	Beta-ketoacyl-ACP reductase
LCA_RS02325	−1.58	NAD(P)-dependent dehydrogenase
LCA_RS04135	−1.54	Beta-hydroxyacyl-ACP dehydratase
LCA_RS03600	−1.69	Glycerate kinase
LCA_RS05480	−1.32	Folylpolyglutamate synthase
LCA_RS09375	−1.04	TVP38/TMEM64 family protein
LCA_RS05495	1.38	Dihydroneopterin aldolase
LCA_RS05475	−1.48	Dihydropteroate synthase
LCA_RS05000	−1.31	Dihydrofolate reductase

## Data Availability

The original contributions presented in the study are included in the article/Supplementary Material; further inquiries can be directed to the corresponding authors.

## Supplementary Materials

The following supporting information can be downloaded at: https://www.mdpi.com/article/10.3390/ijms25137185/s1.
