# Supplementary material for: Transcriptome Analysis Reveals the Role of Sucrose in the Production of Latilactobacillus sakei L3 Exopolysaccharide
Source: Int J Mol Sci. 2024 Jun 29;25(13):7185. doi: 10.3390/ijms25137185 (PMC11241291; doi:10.3390/ijms25137185)
Supplement: Supplementary file 1 [file ijms-25-07185-s001.zip › ijms-3064283-supplementary.pdf]

Transcriptome Analysis Reveals the Role of Sucrose in the Production of *Latilactobacillus sakei* L3 Exopolysaccharide

Binbin wang [1,2,*,†], Baomei Wu [1,†], Min Xu [2], Kaiyue Zuo [1], Ye Han [2] and Zhijiang Zhou [2,*]

[1] School of Life Sciences, Shanxi Normal University, Taiyuan, 030000, China; wubaomei@sxnu.edu.cn (B.W.); zky3092@163.com (K.Z.)

[2] School of Chemical Engineering and Technology, Tianjin University, Tianjin 300072, China; minxu@tju.edu.cn (M.X.); hanye@tju.edu.cn (Y.H.)

* Correspondence: binbinwang@sxnu.edu.cn (B.W.); zzj@tju.edu.cn (Z.Z.)

† These authors contributed equally to this work

Table S1. Differential expression ncRNA and its target genes

ncRNA-ID	GeneID	ncRNA-ID	GeneID	ncRNA-ID	GeneID
		BGI_novel_N140	LCA_RS01020	BGI_novel_N245	LCA_RS06515
		BGI_novel_N140	LCA_RS06515	BGI_novel_N245	LCA_RS09195
BGI_novel_N028	LCA_RS09110	BGI_novel_N140	LCA_RS07025	slsa1083.1	LCA_RS08960
BGI_novel_N028	LCA_RS03775	BGI_novel_N140	LCA_RS03075	slsa1083.1	LCA_RS07880
BGI_novel_N028	LCA_RS07410	BGI_novel_N140	LCA_RS04030	slsa1083.1	LCA_RS01685
BGI_novel_N028	LCA_RS01530	BGI_novel_N154	LCA_RS08485	slsa1083.1	LCA_RS00880
BGI_novel_N058	LCA_RS08485	BGI_novel_N154	LCA_RS09110	slsa1083.1	LCA_RS09110
BGI_novel_N058	LCA_RS00880	BGI_novel_N154	LCA_RS00865	slsa1083.1	LCA_RS00310
BGI_novel_N058	LCA_RS09110	BGI_novel_N154	LCA_RS01515	slsa1083.1	LCA_RS01795
BGI_novel_N058	LCA_RS02295	BGI_novel_N154	LCA_RS01470	slsa1083.1	LCA_RS06435
BGI_novel_N058	LCA_RS01795	BGI_novel_N154	LCA_RS05985	slsa1083.1	LCA_RS03290
BGI_novel_N058	LCA_RS01530	BGI_novel_N154	LCA_RS09195	slsa937.1	LCA_RS08420
BGI_novel_N058	LCA_RS02305	BGI_novel_N154	LCA_RS02665	slsa937.1	LCA_RS01655
BGI_novel_N058	LCA_RS05985	BGI_novel_N187	LCA_RS01875	slsa937.1	LCA_RS08485
BGI_novel_N065	LCA_RS08960	BGI_novel_N187	LCA_RS00970	slsa937.1	LCA_RS05765
BGI_novel_N065	LCA_RS00970	BGI_novel_N187	LCA_RS07875	slsa937.1	LCA_RS00865
BGI_novel_N065	LCA_RS08485	BGI_novel_N187	LCA_RS01655	slsa937.1	LCA_RS00310
BGI_novel_N065	LCA_RS07840	BGI_novel_N187	LCA_RS05240	slsa937.1	LCA_RS01795
BGI_novel_N065	LCA_RS00310	BGI_novel_N187	LCA_RS07450	slsa937.1	LCA_RS09195
BGI_novel_N065	LCA_RS06515	BGI_novel_N187	LCA_RS08485	BGI_novel_N028	LCA_RS09375
BGI_novel_N065	LCA_RS01530	BGI_novel_N187	LCA_RS02310	BGI_novel_N028	LCA_RS04545
BGI_novel_N065	LCA_RS01470	BGI_novel_N187	LCA_RS07345	BGI_novel_N028	LCA_RS06700
BGI_novel_N065	LCA_RS05825	BGI_novel_N187	LCA_RS01795	BGI_novel_N028	LCA_RS07685
BGI_novel_N065	LCA_RS05985	BGI_novel_N187	LCA_RS07410	BGI_novel_N028	LCA_RS05480
BGI_novel_N065	LCA_RS09195	BGI_novel_N187	LCA_RS01515	BGI_novel_N028	LCA_RS06535
BGI_novel_N065	LCA_RS05840	BGI_novel_N187	LCA_RS01530	BGI_novel_N028	LCA_RS05535
BGI_novel_N074	LCA_RS05240	BGI_novel_N187	LCA_RS04405	BGI_novel_N028	LCA_RS03565
BGI_novel_N074	LCA_RS08485	BGI_novel_N187	LCA_RS01470	BGI_novel_N028	LCA_RS03345
BGI_novel_N074	LCA_RS02310	BGI_novel_N187	LCA_RS06805	BGI_novel_N028	LCA_RS04555
BGI_novel_N074	LCA_RS07840	BGI_novel_N187	LCA_RS09195	BGI_novel_N028	LCA_RS06705
BGI_novel_N074	LCA_RS02425	BGI_novel_N187	LCA_RS05840	BGI_novel_N028	LCA_RS03600
BGI_novel_N074	LCA_RS09110	BGI_novel_N232	LCA_RS08485	BGI_novel_N058	LCA_RS09375
BGI_novel_N074	LCA_RS06515	BGI_novel_N232	LCA_RS00880	BGI_novel_N058	LCA_RS05560
BGI_novel_N074	LCA_RS03250	BGI_novel_N232	LCA_RS03775	BGI_novel_N058	LCA_RS08520
BGI_novel_N132	LCA_RS08960	BGI_novel_N232	LCA_RS00865	BGI_novel_N058	LCA_RS07365
BGI_novel_N132	LCA_RS00880	BGI_novel_N232	LCA_RS03280	BGI_novel_N058	LCA_RS06700
BGI_novel_N132	LCA_RS00310	BGI_novel_N232	LCA_RS07300	BGI_novel_N058	LCA_RS05245
BGI_novel_N132	LCA_RS06515	BGI_novel_N232	LCA_RS06515	BGI_novel_N058	LCA_RS02750
BGI_novel_N132	LCA_RS03300	BGI_novel_N232	LCA_RS01470	BGI_novel_N058	LCA_RS06535
BGI_novel_N140	LCA_RS01655	BGI_novel_N232	LCA_RS09195	BGI_novel_N058	LCA_RS04125
BGI_novel_N140	LCA_RS09030	BGI_novel_N232	LCA_RS03250	BGI_novel_N058	LCA_RS02325
BGI_novel_N140	LCA_RS07735	BGI_novel_N245	LCA_RS08485	BGI_novel_N058	LCA_RS04120
BGI_novel_N140	LCA_RS05980	BGI_novel_N245	LCA_RS01795	BGI_novel_N058	LCA_RS03600

ncRNA-ID	GeneID	ncRNA-ID	GeneID	ncRNA-ID	GeneID
BGI_novel_N058	LCA_RS04105	BGI_novel_N132	LCA_RS02790	BGI_novel_N187	LCA_RS01825
BGI_novel_N058	LCA_RS09370	BGI_novel_N132	LCA_RS03360	BGI_novel_N187	LCA_RS05090
BGI_novel_N058	LCA_RS03365	BGI_novel_N132	LCA_RS04555	BGI_novel_N187	LCA_RS02815
BGI_novel_N065	LCA_RS00445	BGI_novel_N132	LCA_RS01590	BGI_novel_N187	LCA_RS01660
BGI_novel_N065	LCA_RS09375	BGI_novel_N132	LCA_RS08235	BGI_novel_N187	LCA_RS05245
BGI_novel_N065	LCA_RS04115	BGI_novel_N132	LCA_RS02325	BGI_novel_N187	LCA_RS02750
BGI_novel_N065	LCA_RS06700	BGI_novel_N132	LCA_RS03475	BGI_novel_N187	LCA_RS00405
BGI_novel_N065	LCA_RS03535	BGI_novel_N132	LCA_RS03600	BGI_novel_N187	LCA_RS04905
BGI_novel_N065	LCA_RS01845	BGI_novel_N132	LCA_RS03355	BGI_novel_N187	LCA_RS09625
BGI_novel_N065	LCA_RS04225	BGI_novel_N140	LCA_RS09375	BGI_novel_N187	LCA_RS03365
BGI_novel_N065	LCA_RS05480	BGI_novel_N140	LCA_RS05690	BGI_novel_N232	LCA_RS09300
BGI_novel_N065	LCA_RS05535	BGI_novel_N140	LCA_RS07365	BGI_novel_N232	LCA_RS00260
BGI_novel_N065	LCA_RS03565	BGI_novel_N140	LCA_RS03490	BGI_novel_N232	LCA_RS05365
BGI_novel_N065	LCA_RS08235	BGI_novel_N140	LCA_RS05970	BGI_novel_N232	LCA_RS05530
BGI_novel_N065	LCA_RS02325	BGI_novel_N140	LCA_RS05245	BGI_novel_N232	LCA_RS03490
BGI_novel_N065	LCA_RS00775	BGI_novel_N140	LCA_RS05480	BGI_novel_N232	LCA_RS05480
BGI_novel_N065	LCA_RS06705	BGI_novel_N140	LCA_RS07095	BGI_novel_N232	LCA_RS07095
BGI_novel_N065	LCA_RS03600	BGI_novel_N140	LCA_RS04555	BGI_novel_N232	LCA_RS04780
BGI_novel_N065	LCA_RS03355	BGI_novel_N140	LCA_RS06705	BGI_novel_N232	LCA_RS04120
BGI_novel_N065	LCA_RS03365	BGI_novel_N140	LCA_RS00990	BGI_novel_N232	LCA_RS09190
BGI_novel_N074	LCA_RS03865	BGI_novel_N140	LCA_RS00555	BGI_novel_N232	LCA_RS01005
BGI_novel_N074	LCA_RS00445	BGI_novel_N154	LCA_RS09300	BGI_novel_N232	LCA_RS00660
BGI_novel_N074	LCA_RS09375	BGI_novel_N154	LCA_RS09375	BGI_novel_N232	LCA_RS03355
BGI_novel_N074	LCA_RS01315	BGI_novel_N154	LCA_RS08640	BGI_novel_N232	LCA_RS02545
BGI_novel_N074	LCA_RS03535	BGI_novel_N154	LCA_RS04115	BGI_novel_N245	LCA_RS09300
BGI_novel_N074	LCA_RS00255	BGI_novel_N154	LCA_RS04915	BGI_novel_N245	LCA_RS03865
BGI_novel_N074	LCA_RS05465	BGI_novel_N154	LCA_RS03720	BGI_novel_N245	LCA_RS00445
BGI_novel_N074	LCA_RS05480	BGI_novel_N154	LCA_RS09505	BGI_novel_N245	LCA_RS09375
BGI_novel_N074	LCA_RS03360	BGI_novel_N154	LCA_RS09335	BGI_novel_N245	LCA_RS01825
BGI_novel_N074	LCA_RS09320	BGI_novel_N154	LCA_RS05245	BGI_novel_N245	LCA_RS07365
BGI_novel_N074	LCA_RS07040	BGI_novel_N154	LCA_RS07685	BGI_novel_N245	LCA_RS08640
BGI_novel_N074	LCA_RS03600	BGI_novel_N154	LCA_RS05480	BGI_novel_N245	LCA_RS01065
BGI_novel_N074	LCA_RS03355	BGI_novel_N154	LCA_RS02750	BGI_novel_N245	LCA_RS06670
BGI_novel_N074	LCA_RS00555	BGI_novel_N154	LCA_RS04610	BGI_novel_N245	LCA_RS03535
BGI_novel_N132	LCA_RS00170	BGI_novel_N154	LCA_RS03360	BGI_novel_N245	LCA_RS00265
BGI_novel_N132	LCA_RS09375	BGI_novel_N154	LCA_RS04555	BGI_novel_N245	LCA_RS04480
BGI_novel_N132	LCA_RS09285	BGI_novel_N154	LCA_RS01060	BGI_novel_N245	LCA_RS05480
BGI_novel_N132	LCA_RS03535	BGI_novel_N154	LCA_RS07370	BGI_novel_N245	LCA_RS06580
BGI_novel_N132	LCA_RS04480	BGI_novel_N154	LCA_RS04135	BGI_novel_N245	LCA_RS05535
BGI_novel_N132	LCA_RS05480	BGI_novel_N154	LCA_RS06705	BGI_novel_N245	LCA_RS03345
BGI_novel_N132	LCA_RS02750	BGI_novel_N154	LCA_RS00990	BGI_novel_N245	LCA_RS05475
BGI_novel_N132	LCA_RS05535	BGI_novel_N154	LCA_RS01370	BGI_novel_N245	LCA_RS08235
BGI_novel_N132	LCA_RS07095	BGI_novel_N187	LCA_RS02985	BGI_novel_N245	LCA_RS08540

ncRNA-ID	GeneID	ncRNA-ID	GeneID	ncRNA-ID	GeneID
BGI_novel_N245	LCA_RS07370	slsa1083.1	LCA_RS05245	slsa937.1	LCA_RS00170
BGI_novel_N245	LCA_RS03475	slsa1083.1	LCA_RS04485	slsa937.1	LCA_RS09300
BGI_novel_N245	LCA_RS04120	slsa1083.1	LCA_RS07685	slsa937.1	LCA_RS06550
BGI_novel_N245	LCA_RS03600	slsa1083.1	LCA_RS01845	slsa937.1	LCA_RS01315
BGI_novel_N245	LCA_RS09190	slsa1083.1	LCA_RS05535	slsa937.1	LCA_RS03535
BGI_novel_N245	LCA_RS09370	slsa1083.1	LCA_RS07370	slsa937.1	LCA_RS07685
BGI_novel_N245	LCA_RS03365	slsa1083.1	LCA_RS09625	slsa937.1	LCA_RS01395
BGI_novel_N245	LCA_RS05470	slsa1083.1	LCA_RS07040	slsa937.1	LCA_RS02750
BGI_novel_N245	LCA_RS04790	slsa1083.1	LCA_RS04760	slsa937.1	LCA_RS08235
BGI_novel_N245	LCA_RS09350	slsa1083.1	LCA_RS03600	slsa937.1	LCA_RS07370
slsa1083.1	LCA_RS09375	slsa1083.1	LCA_RS06880	slsa937.1	LCA_RS09190
slsa1083.1	LCA_RS06550	slsa1083.1	LCA_RS03355	slsa937.1	LCA_RS04800
slsa1083.1	LCA_RS01065	slsa1083.1	LCA_RS03365	slsa937.1	LCA_RS03365

Table S2. Primer sequences of RT-qPCR

Gene ID or name	Forward	Reverse
LCA_RS05240	ACAGAATCACAAGCAAGCGA	CCACCGACAACGAATGGTAA
LCA_RS08985	TGCACCTAAGAGCATCCCTA	CTACTTCTGGGGTAGCTGGT
LCA_RS08990	TTGATGACGGCTTTGACTGT	GCCCAACCGTCTTTATCAGT
16S	GGACGAAAGTCTGATGGAGC	TCTGGTTGGATACCGTCACT

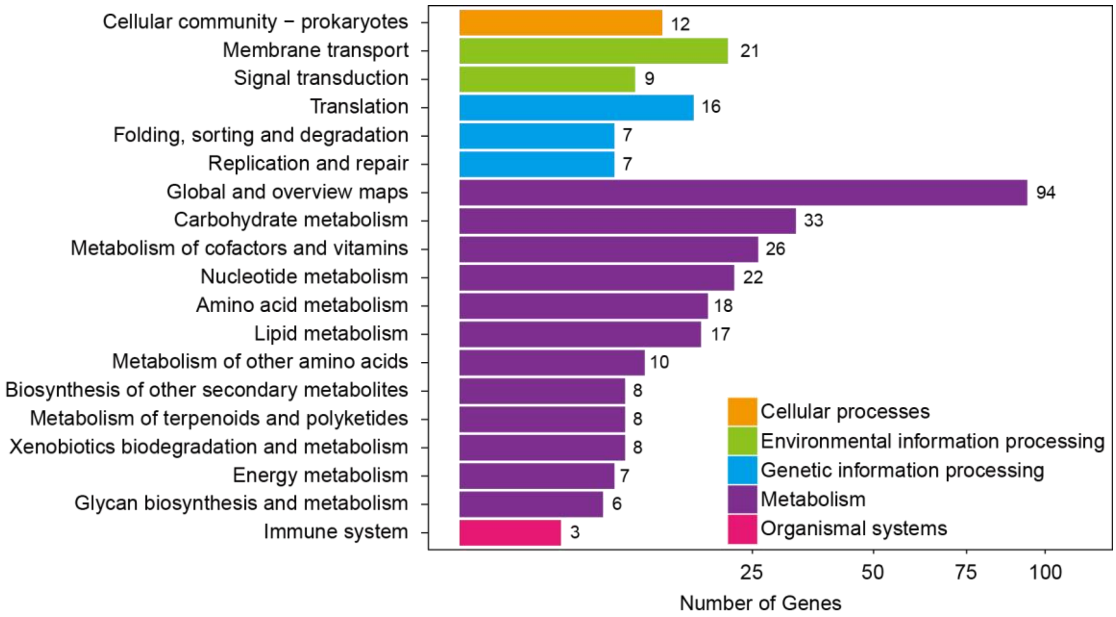

Figure S1. KEGG pathway annotation of DEGs.

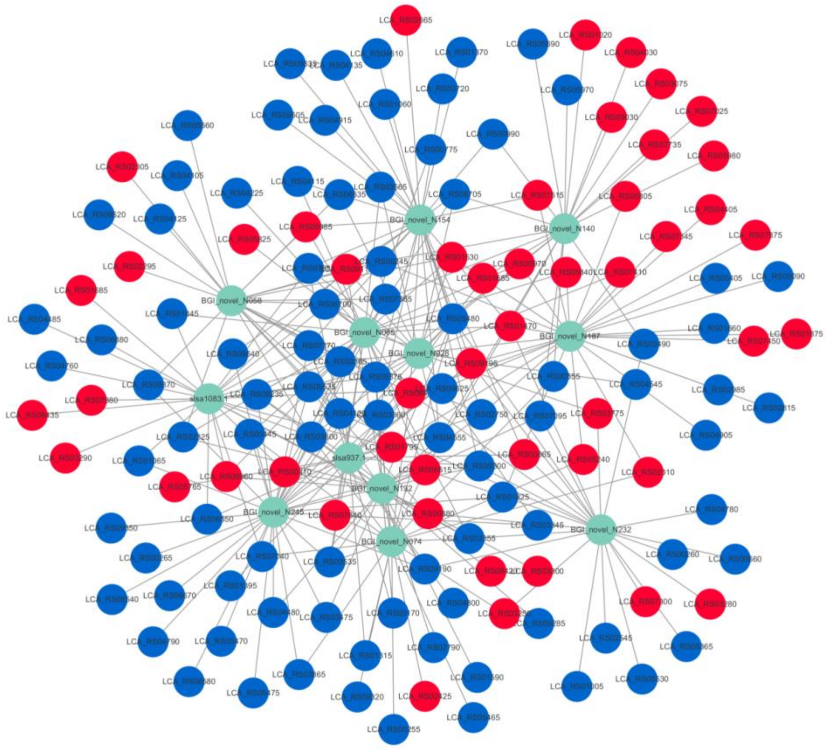

Figure S2. Network of 12 significant differently expressed ncRNAs and their target genes. Red: up-regulated gene, Dark blue: down-regulated gene, Green: 12 ncRNAs
